# Supplementary material for: Use of a specific set of learner-centered evidence-based teaching practices correlates with higher exam performance across seven STEM departments
Source: PLoS One. 2026 Mar 20;21(3):e0327269. doi: 10.1371/journal.pone.0327269 (PMC13004365; doi:10.1371/journal.pone.0327269)
Supplement: S1 Appendix — (PDF) [file pone.0327269.s001.pdf]

Observer ___________ Date of Observation: _______________________ Page ___ of _____

School: _______________ Course: ______________ Instructor: ________________ Session Date: ___________

1. Start of Class (Min:Sec): _______________ 2. End of Class (Min:Sec): _____________

	**Observations:**		**Activity ___**	**Activity ___**	**Activity ___**	**Activity ___**	**Activity ___**
**Intro**	3. Bloom's Level of Activity:		H L	H L	H L	H L	H L
	4. Form of Activity/Question:		MCQ W<br>One SA	MCQ W<br>One SA	MCQ W<br>One SA	MCQ W<br>One SA	MCQ W<br>One SA
**SE: Iteration 1**	5. Start (min:sec):						
	6. End (min:sec):						
	7. Question discussed (Type of SE):		I SG	I SG	I SG	I SG	I SG
	8. Voting included: Yes (Y), No (N)		Y N	Y N	Y N	Y N	Y N
	9. Is the correct answer in any way indicated?		Y N	Y N	Y N	Y N	Y N
**SE: Iteration 2**	10. Start (min:sec):						
	11. End (min:sec):						
	12. Question discussed (Type of SE):		I SG	I SG	I SG	I SG	I SG
	13. Voting included: Yes (Y), No (N)		Y N	Y N	Y N	Y N	Y N
	14. Is the correct answer in any way indicated?		Y N	Y N	Y N	Y N	Y N
**SE: Iteration 3**	15. Start (min:sec):						
	16. End (min:sec):						
	17. Question discussed (Type of SE):		I SG	I SG	I SG	I SG	I SG
	18. Voting included: Yes (Y), No (N)		Y N	Y N	Y N	Y N	Y N
	19. Is the correct answer in any way indicated?		Y N	Y N	Y N	Y N	Y N
**Debrief**	20. Start of Debrief (min:sec):						
	21. End of Debrief (min:sec):						
	22. Who gives the answer?	Instructor (Y/N)					
		# of Volunteers					
		# of Random Calls					
		# of Whole Class					
	23. Who gives the explanation?	Instructor (Y/N)					
		# of Volunteers					
		# of Random Calls					
		No Explanation (N)					
	24. Length of instructor time for teaching/answering/explaining during debrief (min:sec):						
**Activity Characteristics: Can occur at any time**	25. Focus on **logic** (# of times):						
	26. Number of **alternative (or wrong) answers explained**:						
	27. How many times students **asked Qs** to the WC **during activity**?						
	28. **Positive feedback** (# of times):	Entire Class (C)					
		Individual Student (S)					
	29. **Negative feedback** (# of times): C/S						
	30. **Prior knowledge** (# of times):						
	31. Praise **effort/improvement** over ability (# of times):						
	32. **Errors are natural and useful/educational** (# of times):						
	33. How many **questions outside of activity**?						
	34. Comments:						
