# Supplementary material for: Use of a specific set of learner-centered evidence-based teaching practices correlates with higher exam performance across seven STEM departments
Source: PLoS One. 2026 Mar 20;21(3):e0327269. doi: 10.1371/journal.pone.0327269 (PMC13004365; doi:10.1371/journal.pone.0327269)
Supplement: S2 Appendix — (PDF) [file pone.0327269.s002.pdf]

Observer ___________ Date of Observation: ______________________ Page ___ of _____

School: _______________ Course: ______________ Instructor: ________________ Session Date: ___________

1. Start of Class (Min:Sec): _______________ 2. End of Class (Min:Sec): _____________

	Observations:	Activity ___
Introduction	3. Bloom's Level of Activity:<br>Higher order (H), Lower order (L)	High: Analysis, Synthesis, or Evaluation<br>Low: Knowledge, Comprehension, or Application
	4. Form of Activity/Question: Multiple Choice Question (MCQ), Worksheet (W), One word answer (One), Short Answer (SA)	MCQ W<br>One SA
Student Engagement: Iteration 1	5. Start (min:sec):	<u>Students begin to address a question</u> (quietly or audibly).<br>The instructor is not talking, has already asked the question.
	6. End (min:sec):	<u>Instructor starts talking again.</u><br>They could change the mode of the engagement (i.e., from working alone to talk to your neighbors).<br>Ask for feedback (So what did you put?)<br>Begin to explain the answer.
	7. Question discussed (Type of Student Engagement): Individually (I), Small Groups (SG)	Thinking, writing, or answering on their own (Individually).<br>Talking to each other (Small groups).<br>If students seem to be engaging in different ways, record how most are engaging with the activity.
	8. Voting included: Yes (Y), No (N)	Y, N
	9. Is the correct answer in any way indicated? Yes (Y), No (N)	Y, N<br>If the instructor mentions results, displays a histogram of the distribution of student answers to a clicker question, or guides the students with any sort of **hint** to the correct answer, mark as yes. No guidance = no.<br>This is between iteration 1 and 2/debrief.
SE: Iteration 2	10. Start (min:sec):	Occurs if students are given another opportunity to engage in the same activity.<br>Often coincides with a change in the mode of SE (from individual to small group).<br>If the correct answer has been confirmed by the instructor, then it is no longer student engagement, but a **debrief**.
	11. End (min:sec):	
	12. Question discussed (Type of Student Engagement): Individual (I), Small Groups (SG)	I, SG
	13. Voting included: Yes (Y), No (N)	Y, N
	14. Is the correct answer in any way indicated between iterations? Yes, No	Y, N<br>Same as #9.
SE 3: Iteration 3	15. Start (min:sec):	
	16. End (min:sec):	

	17. Question discussed (Type of Student Engagement): Individual (I), Small Groups (SG)	I, SG
	18. Voting included: Yes (Y), No (N)	Y, N
	19. Is the correct answer in any way indicated between iterations?	Y, N
**Debrief**	20. Start of Debrief (min:sec):	<u>Begins when the instructor and students are working towards (discussing) the answer.</u><br>May include explanation of correct answer, alternative answer(s), and/or wrong answer(s).<br>If the instructor asks another question during debrief and students answer the question **without** having time to think alone/do some small group work, then it is coded as part of the **debrief** under the same activity.<br>If the instructor asks the *same question* during debrief and gives students time to think alone or do small group work, then it should be coded as another iteration under the **same activity**.<br>If the instructor asks a *new question* during debrief and gives students time to think alone or do small group work, it is coded as a **new activity**.
	21. End of Debrief (min:sec):	Conversation/discussion about the activity ends.<br>Instructor moves on to new content or a new question/activity.
	22. Who gives the answer?<br>Instructor (Y/N), # of Volunteers (V), # of Random Calls (R), or # of Whole Class (WC)	I (Yes/No)<br>V: R:<br>**Volunteer**: Student raised hand to be called on or can clearly tell that one student has responded to instructor's question.<br>**Random call**: Being called on by the instructor without raising hand, may or may not use randomized list of names.<br>WC:
	23. Who gives the explanation?<br>Instructor (Y/N), # of Volunteers (V), Random Calls (R) or no explanation (N)	I (Yes/No)<br>V: R:<br>Count volunteers/random calls as explaining logic if the student seems to give more than a one- or two-word answer, or you can clearly hear them explaining their logic.<br>N
	24. Length of **instructor time** for teaching/answering/explaining during debrief (min:sec):	**Instructor time**: Instructors are teaching something, which includes answering a question/explaining the answer.<br>**Student time**: when instructors repeat/paraphrase/write the questions, repeat the student's answer, look at list and call student's name, wait for student to respond.<br>Only track instructor time, assume all other debrief time is student time.
**Activity Characteristics**	25. Instances of explicitly encouraging students to **focus on logic** (# of times):	Encourages students to give the reasoning behind their answers.<br>(e.g., "explain/defend your answer", "find evidence that supports your answer", "explain why some answers aren't correct", "could you say more/expand?", "why do you think that?").
	26. Number of **alternative (or wrong answers) explained**:	Number of additional/possible explanations discussed.<br>Number of wrong answer options discussed.<br>Must be more than just a statement (e.g., "This one is wrong." "Not that one.")
	27. How many times students asked questions to the whole class during activity?	Often, these questions come at the very end of the debrief once the question has been answered. The instructor may ask, "Can I answer any questions about this?" In this case, continue the debrief and count the

		number of questions. Amount of time the instructor spends answering the question should be recorded under instructor debrief time.
	28. Instances of explicit **positive** feedback or encouragement (# of times): Directed towards entire class (C), Directed towards individual students (S)	"Nice work!", "I'm impressed!", "You are all doing very well."<br>"Awesome, good work, great job!"<br>Repetitive use of positive expressions (these are all ONE positive feedback).<br><br>NOT: A simple, "Thank you." or "You are correct."<br>Sarcastic positive responses (e.g., "Nice job, most of you got this wrong!").<br>"Better" (because it is implying that work is needed).<br>Expressions for a transitional purpose are NOT positive feedbacks (OK, good - which implies "let's move on the next topic").
	29. Instances of explicit **negativ**e feedback (# of times): Directed towards entire class (C), Directed towards individual students (S)	"That's a stupid answer."<br>"That doesn't make any sense."<br>"You obviously can't do this."<br>NOT: "That is incorrect"
	30. Instances of explicitly encouraging students to use **prior knowledge** (# of times):	Prior knowledge refers to previous day in the same course or previous courses.<br>"Remember what we talked about."<br>"Think back to when we did this before."<br>"You did something similar to this in …class."<br>NOT: Referring to reading for that day's class.
	31. Instances of praise (or encouragement) referencing **effort or improvement over ability** (# of times):	Praising the effort/progress/work students put into the answer/activity.<br>"Wow, you all improved immensely after your discussions."<br>"You obviously worked very hard on that answer."<br>"I'm proud of how much effort you put into this."
	32. Instances of explicitly reminding students that **errors are natural and useful/educational** (# of times):	"Making mistakes is a sign you are learning."<br>"Errors help you develop a better understanding."<br>"If you got everything right, there'd be no reason to be in this class."<br>"Scientists make mistakes too."
	33. How many questions (prompted or unprompted) are asked by students outside of activities?	
	34. Comments:	Technical issues with video, etc.
