# Supplementary material for: Use of a specific set of learner-centered evidence-based teaching practices correlates with higher exam performance across seven STEM departments
Source: PLoS One. 2026 Mar 20;21(3):e0327269. doi: 10.1371/journal.pone.0327269 (PMC13004365; doi:10.1371/journal.pone.0327269)
Supplement: S1 Table — (PDF) [file pone.0327269.s003.pdf]

PORTAAL Code	Long Description	Intensity (I) or Duration (D)	Retained, removed, or combined?
Activities	Number of in-class activities	I	Removed
HB	Number of high Bloom's level activities	I	Retained
MCQ	Number of multiple-choice question activities	I	Removed
WS	Number of worksheet activities	I	Removed
One	Number of one word response activities	I	Removed
SA	Number of short answer question activities	I	Removed
TST	Total amount of time students spent thinking, working, talking, or answering questions	D	Removed
Alone	Number of times students worked alone during activities	I	Retained
SG	Number of times students worked in small groups during activities	I	Retained
Voting	Number of times students voted during activities	I	Removed
Hints	Number of times instructor gave hints during activities	I	Removed
DB	Amount of time in debrief	D	Removed
Ins_Ans	Number of times instructor gave answers during the debriefs	I	Retained
Vol_Ans	Number of times student volunteers gave answers during the debriefs	I	Combined with Vol_Exp
RC_Ans	Number of times randomly called students gave answers during the debriefs	I	Combined with RC_Exp
WC_Ans	Number of times the whole class gave answers during the debriefs	I	Removed
Ins_Exp	Number of times the instructor explained answers during the debriefs	I	Retained

Vol_Exp	Number of times student volunteers explained answers during the debriefs	I	Combined with Vol_Ans
RC_Exp	Number of times randomly called students explained answers during the debriefs	I	Combined with RC_Ans
Exp_Ans	Number of times students explained answers during the debriefs	I	Removed
No_Exp	Number of times no explanation was given by instructor or students during the debrief	I	Removed
ST_DB	Amount of time students spent thinking, working, or answering questions during the debrief	D	Removed
Prom_Log	Number of times the instructor prompted the students to use logic when thinking about or answering a question	I	Retained
Alt_Ans	Number of alternative answers that the instructor or students explained	I	Retained
Act_Q	Number of questions students asked instructor during the activity	I	Removed
Pos_FB_C	Number of times instructor gave positive feedback to the class	I	Combined with Pos_FB_S
Pos_FB_S	Number of times instructor gave positive feedback to an individual student	I	Combined with Pos_FB_C
Neg_FB	Number of times instructor gave negative feedback	I	Removed
PK	Number of times instructor prompted students to use their prior knowledge	I	Removed
Effort	Number of times instructor praised students' effort on an activity	I	Removed
Error_Frame	Number of times instructor told students that errors are useful/educational	I	Removed
Spon_Q	Number of questions students asked instructor not during an activity	I	Removed
