# Supplementary material for: Use of a specific set of learner-centered evidence-based teaching practices correlates with higher exam performance across seven STEM departments
Source: PLoS One. 2026 Mar 20;21(3):e0327269. doi: 10.1371/journal.pone.0327269 (PMC13004365; doi:10.1371/journal.pone.0327269)
Supplement: S2 Table — (PDF) [file pone.0327269.s004.pdf]

Department	Courses		Instructors	Student Data Points
	Total	Introductory		
Biology	25	9	7	4,676
Chemistry	28	24	8	5,532
Computer Science	24	13	6	4,678
Mathematics	12	9	4	1,362
Physics	22	20	8	3,373
Psychology	21	10	5	6,122
Public Health	14	9	7	1,371
**Total**	**146**	**94**	**45**	**27,114**
