# Supplementary material for: Use of a specific set of learner-centered evidence-based teaching practices correlates with higher exam performance across seven STEM departments
Source: PLoS One. 2026 Mar 20;21(3):e0327269. doi: 10.1371/journal.pone.0327269 (PMC13004365; doi:10.1371/journal.pone.0327269)
Supplement: S3 Table — (PDF) [file pone.0327269.s005.pdf]

Quartile	Lower-Division Courses	Upper-Division Courses
Q1	34 (91.9%)	3 (8.1%)
Q2	31 (86.1%)	5 (13.9%)
Q3	24 (66.7%)	12 (33.3%)
Q4	18 (48.6%)	19 (51.4%)
**All quartiles**	**107 (73.3%)**	**39 (26.7%)**
