# Supplementary material for: Use of a specific set of learner-centered evidence-based teaching practices correlates with higher exam performance across seven STEM departments
Source: PLoS One. 2026 Mar 20;21(3):e0327269. doi: 10.1371/journal.pone.0327269 (PMC13004365; doi:10.1371/journal.pone.0327269)
Supplement: S4 Table — (PDF) [file pone.0327269.s006.pdf]

**Demographic Variables**		**Number of Student Data Points**	**Percent of Student Data Points**
Gender	Female	15,298	56.4%
	Male	11,816	43.6%
FGN status	FGN	7,696	28.4%
	Not FGN	19,418	71.6%
EOP status	EOP eligible	5,546	20.5%
	Not EOP eligible	21,568	79.5%
URM status	URM	3,742	13.8%
	Not URM	23,372	86.2%
