# Supplementary material for: Use of a specific set of learner-centered evidence-based teaching practices correlates with higher exam performance across seven STEM departments
Source: PLoS One. 2026 Mar 20;21(3):e0327269. doi: 10.1371/journal.pone.0327269 (PMC13004365; doi:10.1371/journal.pone.0327269)
Supplement: S5 Table — (PDF) [file pone.0327269.s007.pdf]

**Principal Component**	**Quartile (Comparison: Q1)**	**Estimate**	**95% Confidence Interval**	**p-value**
PC1	Quartile 2	0.80	[-0.90, 2.54]	0.4314
	Quartile 3	0.43	[-1.54, 2.39]	0.7297
	Quartile 4	1.79	[-1.31, 4.89]	0.3453
PC2	Quartile 2	3.24	[1.63, 4.84]	0.0003***
	Quartile 3	2.19	[-0.18, 4.56]	0.0899
	Quartile 4	2.03	[-0.89, 4.95]	0.2265

Note. * $p < 0.05$, ** $p < 0.01$, *** $p < 0.001$.

Covariates in the model included students' incoming GPA for each course, gender, EOP status, URM status, first generation status, and course level.
