# Supplementary figures and images for: Use of a specific set of learner-centered evidence-based teaching practices correlates with higher exam performance across seven STEM departments
Source: PLoS One. 2026 Mar 20;21(3):e0327269. doi: 10.1371/journal.pone.0327269 (PMC13004365; doi:10.1371/journal.pone.0327269)

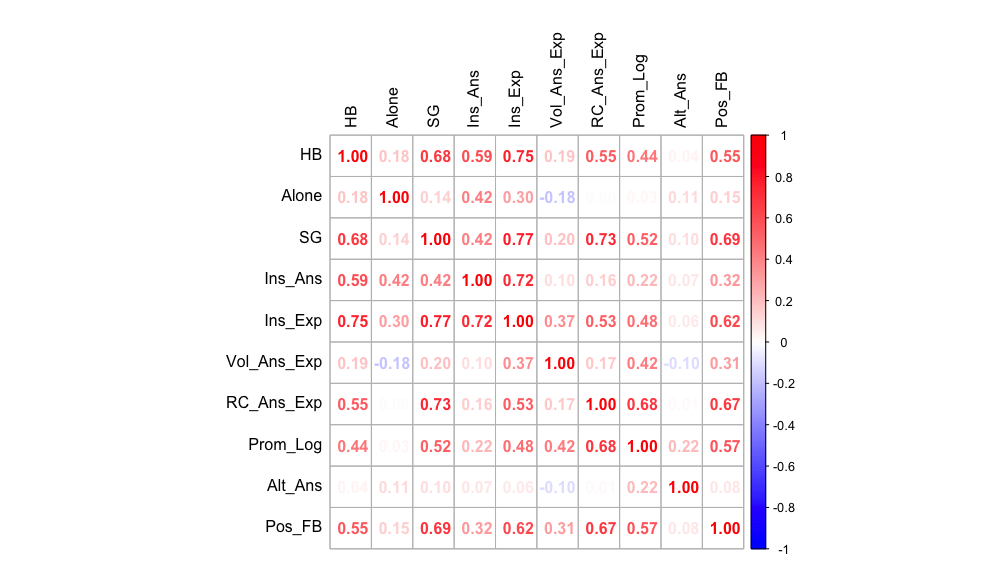

Supplement: S1 Fig — (PNG) [file pone.0327269.s008.png]

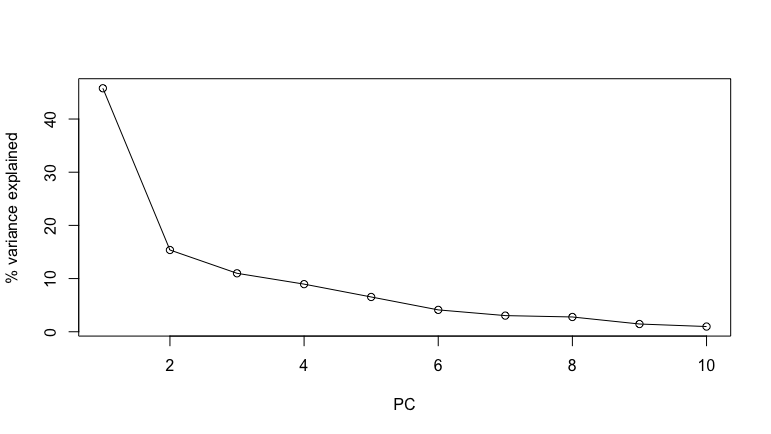

Supplement: S2 Fig — (PNG) [file pone.0327269.s009.png]
